# Effects of simulated altitude (normobaric hypoxia) on cardiorespiratory parameters and circulating endothelial precursors in healthy subjects

**DOI:** 10.1186/1465-9921-8-58

**Published:** 2007-08-08

**Authors:** Michele M Ciulla, Michela Cortiana, Ilaria Silvestris, Emanuela Matteucci, Elisa Ridolfi, Fabrizio Giofrè, Maddalena Zanardelli, Roberta Paliotti, Agostino Cortelezzi, Alberto Pierini, Fabio Magrini, Maria Alfonsina Desiderio

**Affiliations:** 1 Istituto di Medicina Cardiovascolare, Centro Interuniversitario di Fisiologia Clinica e Ipertensione, University of Milan, Ospedale Maggiore Policlinico, Mangiagalli e Regina Elena, Fondazione IRCCS, Via F. Sforza 35 – 20122 Milano, Italy; 2 Dipartimento di Ematologia, Ospedale Maggiore Policlinico, Mangiagalli e Regina Elena, Fondazione IRCCS, Via F. Sforza 35 – 20122 Milano, Italy; 3 Istituto di Patologia Generale, University of Milan, Via L. Mangiagalli, 31 – 20133 Milano, Italy; 4 Istituto di Malattie Respiratorie, University of Milan, Ospedale Maggiore Policlinico, Mangiagalli e Regina Elena, Fondazione IRCCS, Via F. Sforza 35 – 20122 Milano, Italy

## Abstract

**Background:**

Circulating Endothelial Precursors (PB-EPCs) are involved in the maintenance of the endothelial compartment being promptly mobilized after injuries of the vascular endothelium, but the effects of a brief normobaric hypoxia on PB-EPCs in healthy subjects are scarcely studied.

**Methods:**

Clinical and molecular parameters were investigated in healthy subjects (n = 8) in basal conditions (T0) and after 1 h of normobaric hypoxia (T1), with Inspiratory Fraction of Oxygen set at 11.2% simulating 4850 mt of altitude. Blood samples were obtained at T0 and T1, as well as 7 days after hypoxia (T2).

**Results:**

In all studied subjects we observed a prompt and significant increase in PB-EPCs, with a return to basal value at T2. The induction of hypoxia was confirmed by Alveolar Oxygen Partial Pressure (PAO_2_) and Spot Oxygen Saturation decreases. Heart rate increased, but arterial pressure and respiratory response were unaffected. The change in PB-EPCs percent from T0 to T1 was inversely related to PAO_2 _at T1. Rapid (T1) increases in serum levels of hepatocyte growth factor and erythropoietin, as well as in cellular PB-EPCs-expression of Hypoxia Inducible Factor-1α were observed.

**Conclusion:**

In conclusion, the endothelial compartment seems quite responsive to standardized brief hypoxia, possibly important for PB-EPCs activation and recruitment.

## Background

The identification in the peripheral blood (PB) of endothelial precursors (EPCs) derived from bone marrow (BM) and the demonstration of their prompt mobilization, incorporation, and differentiation to the sites of injury have suggested that EPCs could serve as endothelial reparative reserve of the damaged vascular endothelium [1-3]. In addition, in an experimental model of tissue injury it has been demonstrated that, even when injected peripherically, cells derived from BM are able to home to the site of damage [4] contributing to neovessel formation [5]. Therefore, the frequency of PB-ECs has been proposed as diagnostic, therapeutic or prognostic marker of vascular injury and neovascularization [6-9]. Unfortunately, the majority of clinical studies on EPCs focuses on the role of these cells in cardiovascular diseases and no systematic studies exist regarding their variations in healthy subjects, for example under hypoxic conditions. Pathologic tissue ischemia in experimental animal models has been demonstrated to increase the frequency of EPCs, thereby contributing to neovascularization. Cytokines seem to be involved in the mobilization of BM-EPCs [10]. Systemic administration of hepatocyte growth factor (HGF), a multifunctional cytokine involved in tissue repair, induces myocardial angiogenesis which contributes to the improvement in cardiac performance of mice after myocardial infarction [11]. It is known that HGF may exert direct or indirect effects on endothelial cells, also through Vascular Endothelial Growth Factor (VEGF) production [12,13]. The expression of Met, the specific receptor for HGF, is increased in the myocardial infarcted area, where it coexists with CD31, CD34 and WWF-positive cells [11]. The possible role of HGF in activation and recruitment of EPCs in ischemic areas is still unknown. The chemokine SDF-1/CXCL12 and its receptor CXCR4 are critical mediators of the ischemic specific recruitment of circulating EPCs, a loop probably regulated by hypoxia via Hypoxia Inducible Factor-1 (HIF-1) transcription factor activation [14]. HIF-1 is the heterodimeric (α/β) transcription factor that controls tissue oxygen homeostasis [15-17]. The involvement of HGF in the expression of the ligand/receptor couple CXCL12/CXCR4 has not been studied in EPCs, but we have demonstrated that HGF induces CXCR4 in carcinomas [18,19].

Under physiological conditions, exercise is known to upregulate EPCs and to decrease the rate of EPCs apoptosis [20]. Furthermore, *in vitro *induced-anoxia has been shown to enhance the differentiation of peripheral blood mononuclear cells from healthy subjects into EPCs [21]. In a recent paper we have shown that high-altitude hypoxia and exercise oxygen demands are strong stimuli for clonogenic endothelial cell activation [22]. At this regard, no studies are currently available in healthy subjects linking the PB-EPCs response with the hypoxia-specific regulation system. However, hypoxia during ascent to high altitude is responsible for an enhanced expression of Erythropoietin (Epo) and an augment of vascular tone closely related to the increased serum concentration of endothelin (ET)-1. Epo and ET-1 are known target genes of HIF-1 [23].

The present paper aims to assess the effect of a brief standardized normobaric hypoxia in healthy subjects on the frequency of PB-EPCs, and to evaluate early molecular events implicated in the activation and/or recruitment of these cells. To this purpose, we studied the involvement of HIF-1 transcription factor by measuring the expression of the inducible α-subunit and of HIF-1 target- genes. Based on the knowledge in pathological conditions, we chose genes involved in angiogenesis, such as HGF and ET-1, as well as in EPCs recruitment and in erythropoiesis such as CXCR4 and Epo.

## Methods

### Subjects

We enrolled 8 caucasic male healthy non-smokers, non-obese, normo-cholesterol, normotensive, not currently under pharmacological treatment volunteers between our attendant students. No subject had a history of pulmonary disease or respiratory symptoms and all were native sea level dwellers, who had not been at altitude in the preceding three months. Most were not regular exercisers who took part in a range of activities, mainly football. As normal reference, PB-EPCs values obtained in a previous study were used [24]. The study was approved by the Ethical Committee and the subjects signed a consent form.

### Simulated altitude (normobaric hypoxia)

Subjects set quietly breathing room air for five minutes before breathing an oxygen mixture at 11.2% (corresponding to 4850 m) for 1 h by using an hypoxicator (GO2 Altitude, Biomedtech, Australia).

### Cardiorespiratory parameters

During the experiment the main cardiorespiratory parameters were measured. Recordings of Systolic and Diastolic Pressures (SBP, DBP) and heart rate (HR) were obtained non-invasively (Finapress, Ohmeda, Louisville-CO, USA); a gas analyzer (Cosmed Quark b2, Italy) and an oxygen saturimeter (Envitec, Wismar, G) were used to obtain the following parameters: Inspiratory Fraction of Oxygen (FiO_2_), Alveolar Oxygen Partial Pressure (PAO_2_), Spot Oxygen Saturation (SpO_2_), Respiratory Frequency (RF), Tidal Volume (Vt). All cardiorespiratory parameters were measured continuously except SBP and DBP, that were measured at 10 min time intervals.

### Blood sampling and analysis of Endothelial Precursors

A 10 ml PB-sample was obtained from all subjects at each time studied: before (T0) and at the end of the experimental hypoxia (T1), and after 7 days (T2). The frequency of PB-EPCs, defined as KDR+/CD34+/CD45-, was obtained by Flow Cytometry (FACScan, Becton Dickinson, San Jose, CA) according to a previously described procedure on 100,000 events per sample [24].

### Measurement of HGF, Epo and ET-1 levels in the serum

HGF was measured in human serum with Quantikine Immunoassay Kit (R&D System, Minneapolis, MN) following the manufacturer's instructions. Serum concentrations of soluble Epo and ET-1 were assessed by commercial enzyme-linked immunosorbent assays (R&D System). The normal reference values for Epo were within 3.3–16.6 mIU/ml. For ET-1 the sensitivity was 0.14 pg/ml. All the values were calculated using standard curves generated with specific standards, according to the manufacturer's recommendations [25].

### Immunofluorescence assay

Slides were prepared with EPC cells, obtained with immunobeads CD34+ separation, using a cyto-spin. For fluorescence microscopy, the cells were permeabilised with 0.2 % Triton × 100 and were incubated at room temperature with anti HIF-1α antibody (1:50, Transduction Laboratories, Lexington, KY) for 2 h. Non permeabilised-cells were incubated at room temperature with anti CXCR4 antibody (10 μg/ml, MAB172 R&D System) for 1.5 h. Green fluorescent Alexa Fluor-488 (1:800), used as secondary antibody, was let to react for 1 h in the dark. Nuclear staining was performed with DAPI (1:2000). The coverslips were mounted with Entellan (Merck, Darmstadt, Germany), and the cells were examined with a fluorescence microscope (Nikon Eclipse 80i with digital camera DS5MC) at room temperature in the dark. Images were collected through the specimens at 400 × magnification, using an objective Plan APO VC (N.A. 1.40). Analysis of the images was performed according to a previously described procedure [19].

### Statistical analysis

Data obtained for cardiorespiratory parameters were analyzed using a computer statistical software (SPSS – Rel 6.1.1; SPSS Inc., Chicago, Ill). All the quantitative variables were tested for Gaussian distribution with the Kolmogorov-Smirnov test. Changes in any of the studied variables at each time intervals were tested by ANOVA. The relationship between PB-EPCs changes from T0 to T1 and other variables at T1 was tested by regression analysis. In all cases, p < 0.05 was considered significant.

## Results

### Evaluation of clinical parameters in healthy subjects exposed to normobaric hypoxia

**Figure 1:**
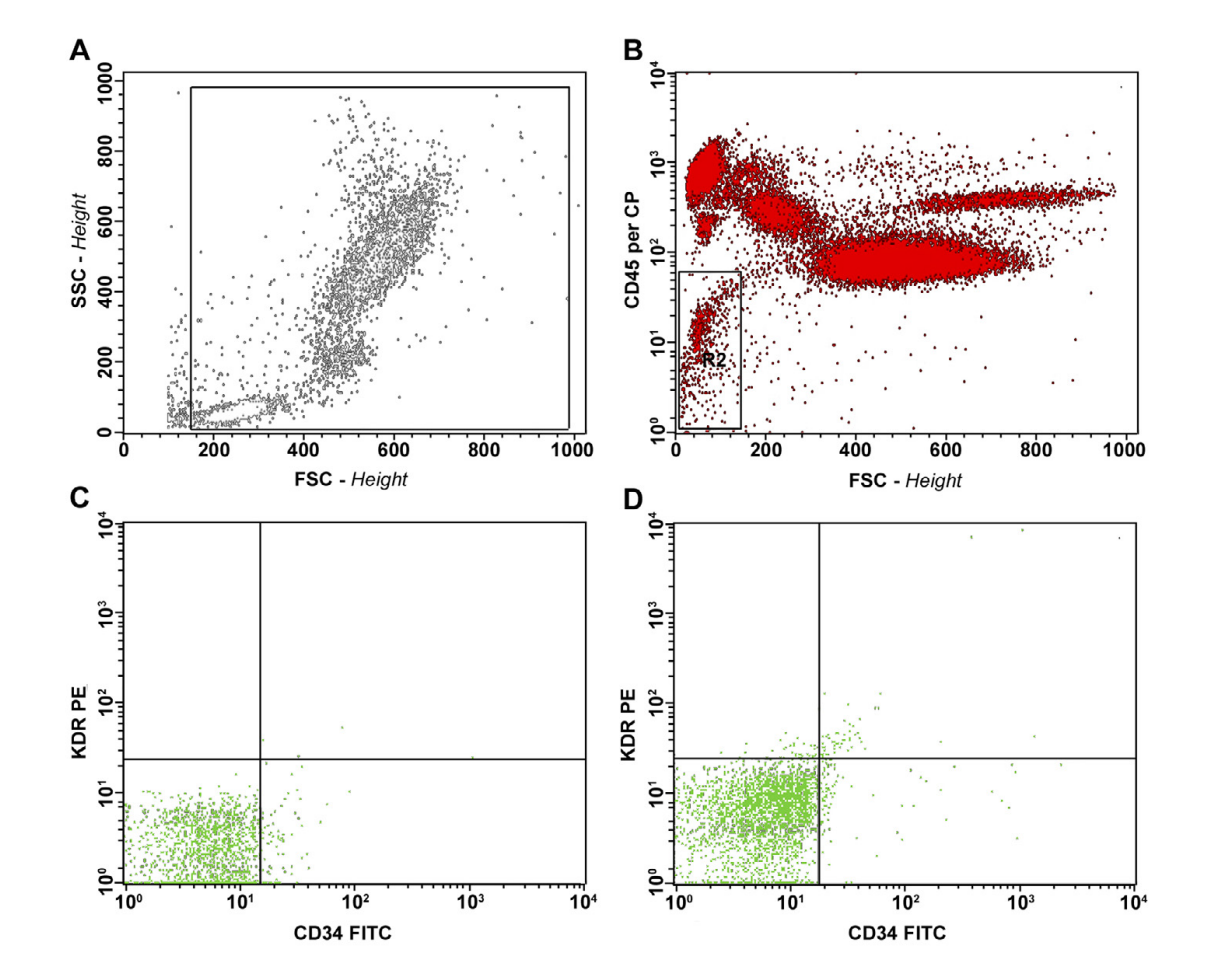
Flow cytometry evaluation of circulating endothelial precursors cells (EPCs). (A) Representative panel showing the analysis gate used to exclude platelets and debris. (B) The gate used to exclude CD45-positive hematopoietic cells. (C, D) Representative panels showing the EPCs before (T0) and after (T1) hypoxia exposure. PerCP, peridin chlorophyll protein; PE, phycoerythrin; FITC, fluorescein isothiocyanate.

**Figure 2:**
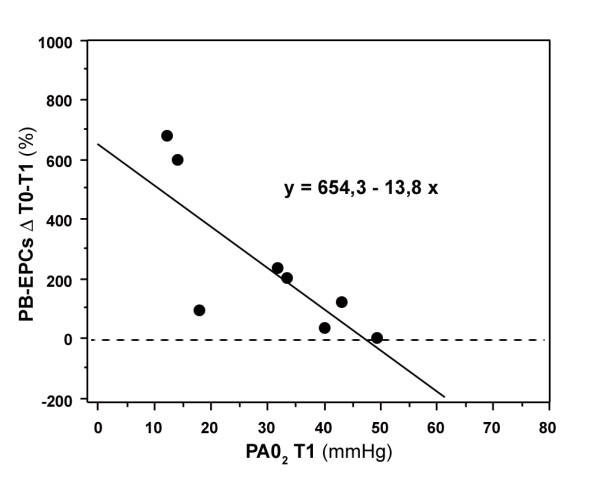
Regression plot showing the correlation between changes in PB-EPCs from T0 to T1 and levels of PAO_2 _at T1 (r = 0.73; p = 0.03). PB-EPCs ΔT0-T1, peripheral blood endothelial precursors change from T0 to T1; PAO_2 _T1, Alveolar Oxygen Partial Pressure at T1.

All subjects completed the test as outlined in the methods section. The induced hypoxia, consisting in an effective FiO_2 _reduction from 20.9 ± 0.5 % to 11.8 ± 0.9 % (Δ- 43.2 ± 5.3%), was confirmed by a significant decrease in PAO_2 _and SpO_2_, respectively, from 104.5 to 30.2 mmHg (p < 0.0001) and from 97.5 % to 86.8 % (p = 0.0005). In all subjects the experimental hypoxia was associated with a prompt and significant increase in the frequency of PB-EPCs, that raised from 0.38 ± 0.56 to 0.65 ± 0.72 cells/ml (p= 0.016) with a return to basal value 7 days after the hypoxia (Table 1). The change in the frequency of PB-EPCs elements was well documented by cytofluorimetric analysis by evaluating CD34+ cells at T0 (Figure 1C) and after hypoxia exposure (T1) (Figure 1D), and excluding CD45+cells (Figure 1B). The significant change in PB-EPCs from T0 to T1 was inversely related to the levels of PAO_2 _at T1 (r = 0.73; p = 0.03), suggesting that PAO_2 _was the trigger of the hypoxia-specific regulation system acting on the endothelial compartment (Figure 2). Furthermore, the gain of the PB-EPCs response was high since a reduction of PAO_2 _from 50 mmHg, where the frequency of PB-EPCs is almost unchanged, to 40 mmHg increased the frequency of PB-EPCs of about 100%.

**Table 1:** Effects of hypoxia on cardiorespiratory parameters and PB-EPCs

	T0	T1	T2	ΔT0-T1%	T0 vs T1 *p*	T1 vs T2 *p*
FiO_2_, %	20.9 ± 0.5	11.8 ± 0.9	21.2 ± 0.5	- 43.2 ± 5.3	< 0.0001	< 0.0001
PAO_2_, mmHg	104.5 ± 14.9	30.2 ± 14.0	99.8 ± 15.8	- 71.8 ± 13.4	< 0.0001	< 0.0001
SpO_2_, %	97.5 ± 1.4	86.8 ± 4.7	96.9 ± 05	- 10.9 ± 5.0	0.0005	0.0005
RF, breaths/min	16.0 ± 2.9	14.4 ± 3.4	15.8 ± 3.0	- 8.3 ± 21.6	0.235	0.118
Vt, L	0.57 ± 0.09	0.56 ± 0.26	0.6 ± 0.2	- 2.2 ± 43.6	0.937	0.426
HR, beats/min	64.0 ± 8.8	77.4 ± 10.8	66.6 ± 10.3	+ 20.9 ± 4.3	< 0.0001	0.002
SBP, mmHg	124.7 ± 4.9	120.3 ± 8.6	122.0 ± 9.0	- 3.5 ± 4.7	0.072	0.645
DBP, mmHg	71.1 ± 3.1	73.0 ± 5.6	76.3 ± 8.2	+ 2.7 ± 8.6	0.418	0.116
PB-EPCs, cells/μL	0.38 ± 0.56	0.65 ± 0.72	0.14 ± 0.20	+ 237.1 ± 264.5	0.016	0.0491

PB-EPCs, peripheral blood endothelial precursors cells; T0. baseline; T1, after 1h of standardized hypoxia; T2, 7 days after hypoxia; FiO_2_, Inspiratory Fraction of Oxygen ; PAO_2, _Alveolar Oxygen Partial Pressure; SpO_2_, Spot Oxygen Saturation; RF, Respiratory Frequency; Vt, Tidal Volume; HR; Heart Rate; SBP, Systolic Blood Pressure; DBP, Diastolic Blood Pressure.

Values are expressed as means ± SD. A p value < 0.05 was considered significant.

In all subjects the cardiac response was characterized by a significant increase in HR (from 64.0 to 77.4 b/min; p < 0.0001) as well as by little reductions in SBP (5/8 subjects) and increases in DBP (5/8 subjects), that did not reach the statistical significance. The pulmonary response was limited, showing little decreases in RF (6/8 subjects) and in Vt (4/8 subjects) not statistically significant (Table 1).

### Oxygen responsive molecules after normobaric hypoxia

**Figure 3:**
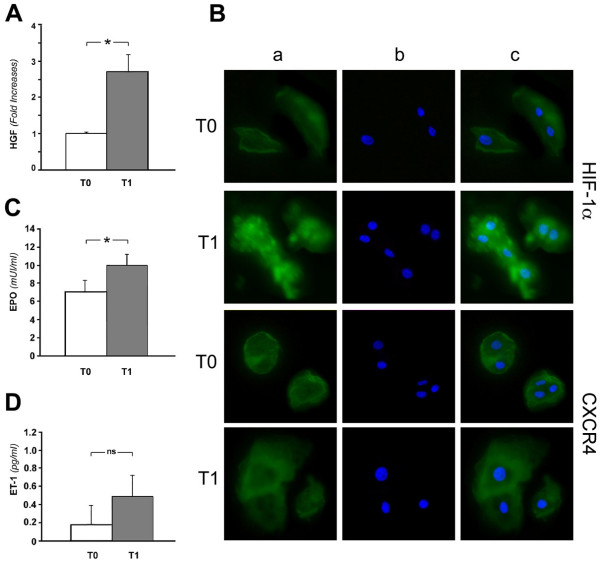
Molecular changes in serum and PB-EPCs after normobaric hypoxia. Normal subjects were examined before (T0) and 1 h (T1) after experimental hypoxia. Serum samples were used for HGF (A), Epo (C) and Et-1 (D) evaluation. HGF data are reported as relative fold-increases, calculated using the absolute values (T0 = 2.9 ± 0.3 ng/ml). All the data were analysed by ANOVA, and the values reported are the means ± S.E. of experiments performed in triplicate. A p value < 0.05 was considered significant. (B) PB-EPCs, prepared on slides, were used to examine HIF-1α and CXCR4 expression by immunofluorescence. Specific stains with anti-HIF-1α or anti-CXCR4 antibody followed by the appropriate secondary antibody (green, a); nuclear staining with DAPI (blu, b); merged image (c). Images were taken using fluorescence microscopy at 400 × magnification.

Baseline levels of HGF (2.9 ± 0.3 ng/ml) were in the normal range reported by the manufacturer; at T1 5/8 subjects showed increases in HGF levels (2.5–2.8-fold relative to T0) (Figure 3A).

The expression of HIF-1α and CXCR4 was assessed by immunofluorescence using PB-EPC, and representative results are shown (Figure 3B). At T1 the expression of HIF-1α protein was strongly enhanced, as shown by the increased fluorescent signal throughout the cell including the nucleus (see merge, c). Stainings with the secondary antibody alone were performed (negative controls), and we did not observe differences in the fluorescence between T0 and T1 (data not shown). Due to the scarce number of PB-EPC collected, it was impossible to perform a quantitative analysis of HIF-1α protein-levels by Western blot. We did not observe changes of CXCR4 protein expression at this early time after hypoxia exposure. After hypoxia a prompt and significant increase in the serum Epo levels was observed in 7/8 subjects included in the experiment, with a change from 7.07 ± 1.04 to 9.91 ± 2.26 mIU/ml (p = 0.009). This increase was directly correlated with the change in PB-EPCs from T0 to T1 (r = 0.65; p < 0.05). A concurrent increase in ET-1 serum levels was observed, that did not reach the statistical significance (from 0.18 ± 0.20 to 0.49 ± 0.58 pg/ml, p = 0.197) (Figure 3C, D).

## Discussion

It is well established that endothelial cells acquire several functional properties in response to diverse extracellular stimuli, and this expression of an altered phenotype is referred to as endothelial cell activation [26]. While it is recognized that endothelial cell activation has a principal role in host defence, recent studies also demonstrate that endothelial cells are capable of complex molecular responses against various forms of stress including hypoxia. Hypoxic stimulus on endothelial cells causes transcriptional induction of genes encoding growth factors for blood vessels and remodelling enzymes [27]. These events are associated with endothelial cell clonogenic activation resulting in loosing/acquiring specific surface markers.

In the present study we have shown that the endothelial compartment promptly reacted to acute hypoxia by increasing the CD34/KDR responsive elements, that are considered PB-EPC. Since KDR is the Fetal liver kinase-1 (Flk-1), i.e. the VEGF receptor, this switch might be functional to the establishment of VEGF-responsive cells [28]. The activation of the PB-EPCs observed was probably related to the systemic release of Epo, and a direct correlation was found between the change in PB-EPCs from T0 to T1 and the serum levels of Epo. The PB-EPCs activation might be mediated by HIF-1α, that is known to regulate the expression of a wide variety of genes involved in neoangiogenesis under various pathophysiological conditions [15,23].

At T1 in PB-EPCs, we observed an enhanced expression of HIF-1α protein, the hypoxia inducible-subunit of the heterodimeric transcription factor HIF-1. Thus, the endothelial compartment seemed to promptly react to a short hypoxia exposure in healthy subjects. Under normoxic conditions, HIF-1α is hydroxylated by prolyl hydroxylases. This reaction enables the von Hippel-Lindau (VHL) protein-binding and HIF-1α degradation by the proteasome. Under hypoxic conditions, prolyl hydroxylases are inactive, HIF-1α is stabilized and translocates to the nucleus as an heterodimer with the constitutive HIF-1β. This control mechanism, i.e. protein stabilization, might be responsible for the rapid enhancement in HIF-1α expression observed after normobaric hypoxia.

The HIF-1 complex binds to hypoxia-responsive elements (HRE) sequences in the promoters of target genes, causing activation of transcription. Under hypoxic conditions, HIF-1 is known to transactivate VEGF, Epo, and ET-1 as well as genes involved in cell recruitment such as the chemokine receptor CXCR4 and the ligand SDF-1/CXCL12 [14,23,29,30]. In our study despite an absolute increase in serum ET-1, the changes observed were not significant. Also, CXCR4 expression at T1 in PB-EPCs was unaffected. The lack of significant changes in ET-1 and CXCR4 was probably related to the short exposure to hypoxia. In fact, molecular events controlled at transcriptional/translational level need longer times to occur. Consistently HGF, a growth factor involved in neoangionenesis like VEGF [12,13], was found increased in the serum of healthy subjects exposed to normobaric hypoxia. HGF is known to be rapidly released in the blood after various types of tissue injuries. Present as a pro-form in tissue extarcellular matrix, HGF is activated and released by proteolytic mechanisms [31]. Thus, HGF seemed to play a critical role in early stages of EPCs activation, and might contribute to the triggering of later molecular events.

Studies are in progress at examining these possible changes dependent on early HIF-1 alpha expression, regarding for example the transcription via HIF-1 of CXCR4 and Met receptors important for cell scattering and homing [11,14]. Met expression possibly enhances the sensitivity to HGF. Also HIF-1 expression, regarding for example CXCR4 and Met receptors important for cell scattering and homing [11,14]. Met expression possibly enhances the sensitivity to HGF. Also HIF-1 might regulate its transcription due to the presence of HREs in the promoter, amplifying therefore the response to hypoxia [32].

The hypoxic cardiorespiratory response is a complex interplay between several distinct mechanisms, and the O_2 _level-decrease is sensed by specialized chemoreceptor cells that regulate cardiovascular and ventilatory response [33]. The normal hemodynamic response to acute hypoxia consists of an increase in HR and a modulation of the vascular tone, with vasodilatation of peripheral vessels and constriction of the vessels of the pulmonary vasculature to shunt blood away from the poorly ventilated region. In our subjects the increase in HR is prompt and prevalent (8/8 subjects), confirming that small reductions in PAO_2 _are detected by peripheral chemoreceptors eliciting the sympathetic chemoreflex [33]. On the ventilatory side, the normal response consists of a gradual increase in ventilation that intensifies over the following hours and days [34]. This ventilatory response was not univocally observed in our subjects probably because the hypoxic exposure was too short to recruit the carotid chemoreceptors [35].

The wide range of oxygen tensions found in healthy tissue makes it difficult to establish an universal value to define hypoxia [36]. Based on the medical definition of high altitude, consisting in an elevation of 2700–5500 m above sea level [33], in our study we have set the FiO_2 _to an altitude of 4850 mt. On the endothelial point of view, the definition of hypoxia could be the level of oxygen at which the clonogenic PB-EPCs response starts. Furthermore, this cellular response might be used for studies aimed to predict the adverse effects of high altitude since it has been shown that limited informations can be obtained by assessing only cardiorespiratory physiological variables at sea level and at a range of simulated altitudes [37].

### Study limitations

On the technical point of view, the main limitations of all studies based on circulating EPCs enumeration consist in the lack of a uniform immunophenotype definition of EPCs and therefore of an experimental method to discriminate between different populations [24]. Furthermore we do not provide any informations on EPCs function such as migration or ability to form colonies; however at this regard we have previously shown that high-altitude hypoxia and exercise is able to switch on the endothelial colony-forming unit capacity [22].

## Conclusion

In conclusion, the cellular and molecular data indicate an evident excess in the response of the endothelial compartment when considering a physiological stimulation. In healthy subjects the frequency of PB-EPCs may be considered as a marker of endothelial activation after exposure to physiological normobaric hypoxia, corresponding to an altitude of 4850 mt, that may be important for neovascularization. This study may contribute to a better understanding of the molecular mechanisms involved in the early steps of the normobaric hypoxia. Even if hypoxia is a potent modulator of gene expression influencing the expression of approximately 1.0% of the genes in the genome [35], the identification of a specific oxygen sensor remains elusive. Studies are in progress to evaluate late events responsible for PB-EPCs homing to hypoxic tissues.

## Competing interests

The author(s) declare that they have no competing interests.

## Authors' contributions

MMC designed research, collected and analyzed data, wrote and critically revised the paper; MAD participated to the design of the research, analyzed data, and critically revised the paper; AP, MC, IS, EM, ER, MZ, and FG performed research; RP analyzed data, wrote and critically revised the paper; AC critically revised the paper; FM critically revised the paper.
